# Nutrition-sensitive agriculture programs increase dietary diversity in children under 5 years: A review and meta-analysis

**DOI:** 10.7189/jogh.12.08001

**Published:** 2022-02-19

**Authors:** Amy Margolies, Christopher G Kemp, Esther M Choo, Carol Levin, Deanna Olney, Neha Kumar, Ara Go, Harold Alderman, Aulo Gelli

**Affiliations:** 1 International Food Policy Research Institute, Washington, D.C., USA; 2 Department of International Health, Johns Hopkins University, Baltimore, Maryland, USA; 3 Department of Global Health, University of Washington, Seattle, Washington, USA

## Abstract

**Background:**

Low-quality diets contribute to the burden of malnutrition and increase the risk of children not achieving their developmental potential. Nutrition-sensitive agriculture programs address the underlying determinants of malnutrition, though their contributions to improving diets do not factor into current nutrition impact modeling tools.

**Objective:**

To synthesize the evidence on the effectiveness of nutrition-sensitive agriculture programs in improving dietary diversity in young children (6-23.9 months and 6-60 months).

**Methods:**

A literature search was conducted for published trials through existing systematic reviews and individual database search of the ISI Web of Science. All dietary diversity measures in the studies selected to be in the analysis were extracted. Estimation of main pooled effects were conducted on outcomes of minimum diet diversity (MDD) and diet diversity score (DDS) using random-effects meta-regression models. We report pooled effect sizes as standardized mean differences (SMDs) or odds ratios (ORs).

**Results:**

Nutrition-sensitive agricultural interventions have a significant positive impact on the diet diversity scores of children aged 6-23.9 months (SMD = 0.22, 95% confidence interval (CI) = 0.09-0.36) and on the odds of reaching minimum diet diversity (OR = 1.45, 95% CI = 1.20, 1.76). Similar impacts are found when analyses are expanded to include studies for children aged 6-60 months (DDS SMD = 0.22, 95% CI = 0.12-0.32) (MDD OR = 1.64, 95% CI:  = 1.38-1.94).

**Conclusion:**

Nutrition-sensitive agriculture interventions consistently have a positive impact on child dietary diversity. Incorporating this evidence in nutrition modeling tools can contribute to decision-making on the relative benefits of nutrition-sensitive interventions as compared with other maternal, newborn, child health and nutrition (MNCHN) interventions.

Estimates of the global burden of disease have attributed 20% of deaths to unhealthy diets [1]. Poverty, malnutrition, and other adversities result in over 250 million children being at risk of not achieving their developmental potential [2]. Multi-sectoral nutrition-sensitive programs aim to address the determinants of nutrition and include nutrition-focused goals or strategies. These interventions may also serve as platforms for scaling nutrition-specific interventions which target the immediate determinants of child nutrition [3]. A growing body of evidence shows that nutrition-sensitive programs can lead to improved nutrition outcomes for children and their mothers [4]. Integrated agriculture and nutrition interventions have been found to consistently increase household access to nutritious foods leading to improvements in the diets of women and young children, but are less likely to directly lead to reductions in child stunting [3,4]. Diet diversity is positively associated with nutrient adequacy – or diets meeting essential nutrient requirements in women and young children [5-7]. Additionally, diet diversity has been found to be positively associated with lower odds of stunting in children 6-23 months [8].

Nutrition-sensitive agricultural programs take a variety of forms. The agricultural components include interventions to support the production of diversified or more nutritious crops, livestock rearing and aquaculture. The nutrition components of these interventions often include behavior communication change (BCC) approaches that promote positive health behaviors such as the diversification of diets or improved child feeding and/or sensitization or trainings to improve women’s empowerment through agriculture.

Although the evidence is clear that nutrition-sensitive agriculture programs can improve children’s diets, the size of the effect is unknown. However, as evidence on these interventions is growing, there is an opportunity to address the question systematically by using a meta-analysis to determine the overall size of the effect. The objective of this study is to synthesize the evidence on the effectiveness of nutrition-sensitive agriculture programs in improving dietary diversity in young children (6-23.9 months and 6-60 months). The results from this meta-analysis can be incorporated into modeling exercises and can serve to inform program design and policy development.

## METHODS

### Search strategy

Our search covered a variety of nutrition-sensitive agriculture interventions. Intervention types ranged from biofortification – using conventional plant breeding methods to produce more nutritious crops – paired with behavior communication change (BCC), to school-based or homestead gardening supported by agricultural training and inputs accompanied with nutrition education. We searched for published trials through the existing reviews cited above and also conducted a database search for articles in all languages in Google Scholar and the ISI Web of Science Core Collection – a unifying research tool that aggregates from multiple databases (Web of Science Core Collection: Citation Indexes includes: 1) Science Citation Index Expanded (SCI-EXPANDED)-1974-Present, 2) Social Sciences Citation Index (SSCI)-1974-present, 3) Arts & Humanities Citation Index (A&HCI)-1975-Present, 4) Emerging Sources Citation Index (ESCI)-2015-present)[9]. We utilized terms to refine our search to evaluations of programs that included at least one nutrition outcome. The search terms included AND nutrition AND several specific diet and nutrition outcomes separated by OR. For those interventions we considered nutrition-sensitive agriculture programs, the more specific search terms included [biofortification OR “agricultur* extension” OR livestock OR (agriculture* AND dairy) OR “home* garden*” OR “homestead food production”]. For diet-related outcomes, we included search terms for diet and infant and young child feeding practices as well as for micronutrient (MN) intake and calorie consumption. For nutrition outcomes, we used search terms for anthropometric measures, anemia and micronutrient status. However, we did not use nutrition outcomes beyond diets in this study.

### Inclusion and Exclusion criteria

The database search yielded a total of 972 publications. We reviewed titles and abstracts from this initial group by sub-topic in order to refine which studies would be fully reviewed for inclusion. Publications were excluded during the title and abstract review if they were review studies, observational studies with only one time point, or were conducted in high-income countries, leaving 20 studies. We also included studies published in recent systematic reviews (Ruel et al., 2018; Sharma et al., 2021) or in peer-reviewed journals between 2018 (the year of the recent systematic review) and the current year. Seventeen studies were included from [4], and 39 studies were added from a new systematic review published after the original literature search had been conducted [10], resulting in 56 records from existing reviews. Duplicates were then removed between the database search and existing reviews, resulting in 37 records. After further review, 11 records did not meet the inclusion criteria and were excluded from this group. This process culminated in a total of 26 publications for full review. The full text articles were then screened using our inclusion and exclusion criteria. Criteria for inclusion in the meta-analysis in addition to the overall quality of the study included: longitudinal designs/quasi-experimental designs/trials with a valid counterfactual (control group) and at least two time points, and trials reporting at least one outcome of diet diversity at the individual level. We then further screened these studies to identify those that specifically measured dietary diversity in young children using the standard WHO definition. Age inclusion criteria included children under five years of age. Initially, we included all potential individual measures of diet diversity across all age groups of children and mothers. In addition, the co-authors reviewed results of the first search and identified and included missing publications based on their expertise. Finally, 9 studies were selected to be included in the quantitative synthesis, 5 from prior reviews and 4 from database searches.

### Data extraction and quality assessment

The data extraction process began with a pilot extraction. One author (CK) created a structured abstraction form. All co-authors reviewed, critiqued and approved the form. Two authors (CK, EC) piloted this form with two studies and made modifications as needed. Then, they assigned all other authors a portion of studies for primary or secondary extraction. Data were extracted in two stages after piloting. First, all co-authors independently extracted general information into the standardized form about study setting, intervention design and inputs, year, country, study type, target population, time period of intervention implementation, study design, treatment arm description, sample size and study population, and outcomes measured. Second, we extracted all dietary diversity measures in these studies. Initially, we included all types of diet diversity measures, from nutrient intake, diet diversity scores (DDS), minimum diet diversity (MDD) and to more narrowly defined indicators such as number of fruits or vegetables consumed or quantity of animal-sourced foods consumed. We also extracted information on the sample size for the outcome measure, effect measure description and value, standard error, 95% CI and statistical significance. Specification of outcomes and effect measures were assessed for comparability across trials. Data were reviewed for accuracy and consistency and all studies were independently dual-coded by the authors. No disagreements for inclusion of studies were encountered between the authors.

### Quantitative data synthesis

We estimated the main pooled effects on outcomes of minimum diet diversity (MDD) and diet diversity score (DDS), two indicators based on counts of food groups consumed over a given period that are widely used to assess dietary diversity in low-income countries [11].

The MDD is a score developed by the World Health Organization (WHO) that assesses diet diversity among children aged 6-23 months as part of infant and young child feeding (IYCF) practices [12]. The MDD is a population-level indicator that measures the number of children in that age group who received foods from at least 5 out of 8 food groups the preceding day from the total children from whom diet data were collected [13]. The indicator has been validated as a proxy for nutrient adequacy for children aged 6-24 months [14] and more recently for children aged 24-59 months [15]. The current MDD measure has been updated to include breast milk as an 8th food category. However, due to the timing of the studies within this review, only the former version of the MDD (minimum 4 out of 7 food groups) was included as an outcome [12]. The MDD indicator is a binary outcome with the principal measure of effect as the odds ratio – the relative difference in odds of the outcome between treatment groups at endline. We used adjusted ORs and 95% confidence intervals (CIs). Where we had no adjusted ORs, we calculated the Ns for a 2x2 table. We then calculated odds and variance.

The diet diversity score (DDS) is a measure of population-level diet diversity that can be measured at household-level to assess household food security status or at the individual level for diet diversity. The DDS specifies the average number of food groups out of seven pre-specified food groups consumed by the target group the prior day. This score has been validated against the micronutrient adequacy of a diet in young children 6-23 months [14] as well as in children 24-60 months of age [6,16]. The principal measure of effect used for this continuous outcome was the mean difference between intervention and control groups at endline. We used the means and standard deviations (SDs) across treatment arms to calculate standardized mean differences (SMD) and variance. In those cases where we were not able to obtain means and SDs across treatment arms but had access to sample sizes in both arms and *P* values, we calculated the t-statistic and used the t-statistic to calculate Cohen’s d.

We stratified our findings by treatment comparison in order to clearly distinguish between those studies with a naïve control (ie, a control that did not receive any intervention), and those studies with a control that received some form of agricultural support (ie, not a naïve control). This distinction allowed us to better understand the implications of the meta-analysis considering the variation among program and study designs.

Effect size estimates were pooled using random-effects meta-regression models. Analyses were conducted using the R software (R Core Team, Vienna, Austria, 2019) [17] and the *metafor* package [18]. We report pooled effect sizes as ORs for effects on the binary MDD measure, and SMDs for effects on the continuous DDS measure. We assessed bias between studies by examining between-study heterogeneity using the Cochrane Q test and *I^2^* proportion. The trim-and-fill method was used to test for publication bias. Effects were pooled across the 6-23.9-month age group in the primary analysis. The 6-23.9-month age group was prioritized as it has been widely validated and because the first 1000 days of a child’s life are the critical period for nutrition interventions [19]. We then expanded our analysis to a broader age group (6-60 months) which allowed us to examine potential effects pooled from a greater number of studies.

## RESULTS

### Trial flow

**Figure 1:**
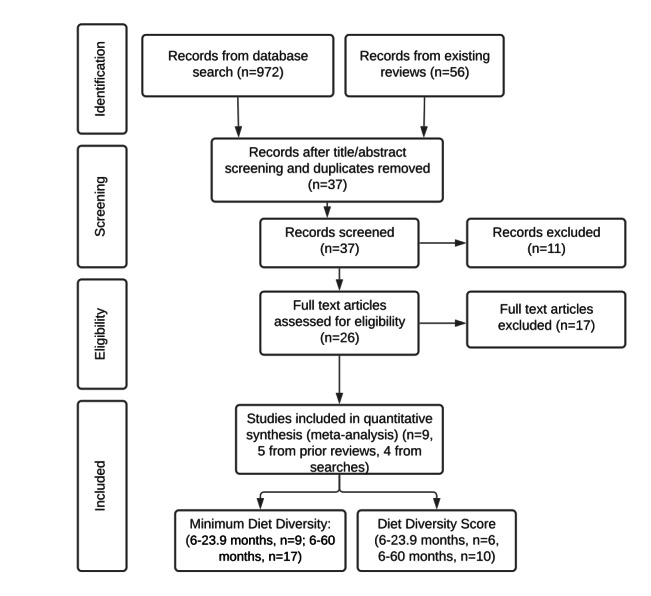
PRISMA flowchart (Preferred Reporting Items for Systematic Reviews and Meta-Analyses).

**Figure 1** presents a PRISMA flowchart showing the full article review process. Nine trials were retrieved for analysis, 5 from prior reviews and 4 new studies from database searches, providing evidence from eight countries: Mozambique, Zambia, Burkina Faso, Malawi, Ethiopia, Ghana, Kenya and Cambodia. All studies were analyzed for the binary MDD outcome for children aged 0-23.9 months, and seven of those trials were also analyzed for the continuous diet diversity score (DDS) outcome. Some studies had more than one treatment group. In these cases, the treatments were entered as a separate row in the analysis: 6-23.9 months, MDD: n = 9 studies and DDS: n = 6 studies; 6-60 months, MDD: n = 17 studies and DDS: n = 10 studies. All studies were deemed evidence of high quality as all had counterfactuals and most were randomized control trials (RCTs) with the exception of [20], which had a control group but was not randomized.

The included articles (n = 9) evaluate a range of nutrition-sensitive agriculture interventions. Most of the programs included in-kind transfers and almost all included other complementary program components such as agriculture trainings or BCC around health and nutrition. The intervention components of the programs included in our analysis are outlined in **Table 1**.

**Table 1:** Intervention components of nutrition-sensitive agriculture programs included in the meta-analysis (n = 9)

Country (reference)	Trial design	Arm	Intervention components					
**Inputs**				**Training**	
**Agricultural tools**	**Seeds/vines**	**Poultry**	**Livestock**	**Health/ nutrition BCC**	**Agriculture**
Malawi (Gelli et al. 2018) [21]	cRCT			×	×		×	×
Ghana (Marquis et al. 2018) [22]	cRCT			×	×		×	×
Burkina Faso (Olney et al. 2015) [23]	cRCT	OWL	×	×	×		×	×
Burkina Faso (Olney et al. 2015) [23]	cRCT	HC	×	×	×		×	×
Ethiopia (Passarelli et al. 2020) [24]	cRCT	ACGG			×			×
Ethiopia (Passarelli et al. 2020) [24]	cRCT	ACGG/ATONU		×	×		×	×
Zambia (Kumar et al. 2018; Rosenberg et al. 2018) [25,26]	cRCT	Ag-G-BCC	×	×	×	×	×	×
Zambia (Kumar et al. 2018) [25]	cRCT	Ag-G	×	×	×	×		×
Mozambique (Low et al. 2007) [20]	QE			×			×	×
Malawi (Kuchenbecker et al. 2017) [27]	cRCT						×	×
Cambodia (Reinbott et al. 2016) [28]	cRCT						×	×
Kenya (Boedecker et al. 2019) [29]	cRCT						×	×

cRCT – cluster randomized controlled trial, QE – quasi-experimental study, BCC – behavior change communication, OWL – older woman leader, HC – health committee, ACGG – African Chicken Genetic Gains, ATONU – agriculture to nutrition, AG-G – agriculture, gender equity and women’s empowerment

Olney et al (2015) [21] evaluated a 2-year integrated agriculture and nutrition and health behavior change communication (BCC) program targeted to women and their children aged 3-12.9 months in Burkina Faso. This program supported the development of school/homestead gardens, provision of information, training and inputs. Low et al (2007) [20] studied an intervention in Mozambique that ran from 2003-2004. The intervention included nutrition education (BCC) and agriculture (agriculture extension training, inputs, participatory nutrition BCC, mass media communication and market linkages) and targeted children under 3 years of age. Marquis et al (2015) [22] assessed an agriculture-nutrition intervention in Ghana that promoted home gardening and poultry farming, providing inputs and nutrition education. Gelli et al (2018) [23] evaluated an agriculture-nutrition program delivered through community-based childcare centers, which included the provision of agricultural inputs and nutrition education. Passarelli et al (2020) [24] assessed a livestock intervention with poultry, including two different treatment arms - considered for our purposes as two different interventions - one with an additional BCC and agricultural input provision component. Kuchenbecker et al (2017) [25] studied a nutrition education and agriculture intervention that included agricultural extension and infant and young child feeding (IYCF) nutrition training in Malawi. Boedecker et al (2019) [26] evaluated a participatory farm diversification and nutrition education program in Western Kenya that centered around kitchen gardens. Kumar et al (2018) [27] assessed a nutrition and gender-sensitive agriculture intervention that provided agricultural inputs including chickens and goats along with IYCF BCC. Similarly, Reinbott et al (2016) [28] evaluated an IYCF-focused nutrition BCC program that promoted improved farming and provided input vouchers in Cambodia.

As a final step, we verified the outcomes of interest and CIs with the original study authors and gathered outstanding measures and sample sizes as necessary to complete our analysis.

### Pooled estimates of effect

**Table 2** and **Table 3** summarize pooled estimates of the effects of different types of nutrition-sensitive agriculture interventions on child diet diversity measures by age of child (6-23.9 months and 6-60 months). These tables show the pooled ORs and SMDs for each indicator and intervention type. SMDs represent the relative effect size in the intervention as compared to a control group.

**Table 2:** Summary of findings for pooled analysis of nutrition-sensitive agriculture interventions on child diet diversity (6-23.9 months)

Outcome of interest	Comparisons	Design	Consistency	Intervention, n	Control, n	OR (95% CI)	SMD (95% CI)
MDD, 3 comparisons	Agriculture + Nutrition BCC vs Control	cRCT	2 studies suggest benefit in OR	724	717	1.72 (1.05, 2.82)	
MDD, 4 comparisons	Agriculture + Nutrition BCC vs Agriculture	cRCT	2 studies suggest benefit in OR	1511	1317	1.47 (1.10, 1.96)	
MDD, 2 comparisons	Agriculture vs Control	cRCT	0 studies suggest benefit in OR	453	570	1.18 (0.91, 1.53)	
**Overall MDD, 9 comparisons**		cRCT	4 studies suggest benefit in OR	2688	2604	1.45 (1.20, 1.76)	
DDS, 2 comparisons	Agriculture + Nutrition BCC vs Control	cRCT	2 studies suggest benefit in SMD	663	645		0.33 (0.02, 0.64)
DDS, 3 comparisons	Agriculture + Nutrition BCC vs Agriculture	cRCT	2 studies suggest benefit in SMD	1450	1264		0.20 (-0.01, 0.41)
DDS, 1 comparison	Agriculture vs Control	cRCT	0 studies suggest benefit in SMD	401	498		0.12 (-0.01, 0.25)
**Overall DDS, 6 comparisons**		cRCT	3 studies suggest benefit in SMD	2514	2407		0.22 (0.09, 0.36)

MDD – Minimum Dietary Diversity, BCC – Behavior Change Communication, DDS – Dietary Diversity Score, OR – odds ratio, SMD – standardized mean difference, QE – quasi-experimental, cRCT – cluster randomized controlled trial

**Table 3:** Summary of findings for pooled analysis of nutrition-sensitive agriculture interventions on child diet diversity (6-60 months)

Outcome of interest	Comparisons	Design	Consistency	Intervention, n	Control, n	Adjusted OR (95% CI)	SMD (95% CI)
MDD, 9 comparisons	Agriculture + Nutrition BCC vs Control	cRCT	8 studies suggest benefit in OR	2053	2001	2.04 (1.58, 2.63)	
MDD, 5 comparisons	Agriculture + Nutrition BCC vs Agriculture	QE/cRCT	2 studies suggest benefit in OR	1706	1529	1.47 (1.16, 1.86)	
MDD, 3 comparisons	Agriculture vs Control	cRCT	0 studies suggest benefit in OR	665	808	1.17 (0.94, 1.45)	
**Overall MDD,** 17 comparisons		QE/cRCT	10 studies suggest benefit in OR	4423	4338	1.64 (1.38, 1.94)	
DDS, 8 comparisons	Agriculture + Nutrition BCC vs Control	QE/cRCT	5 studies suggest benefit in SMD	2303	2179		0.28 (0.16, 0.40)
DDS, 4 comparisons	Agriculture + Nutrition BCC vs Agriculture	cRCT	3 studies suggest benefit in SMD	1705	1527		0.22 (0.06, 0.38)
DDS, 2 comparisons	Agriculture vs Control	cRCT	0 studies suggest benefit in SMD	664	809		-0.01 (-0.28, 0.26)
**Overall DDS,** 10 comparisons		QE/cRCT	7 studies suggest benefit in SMD	4672	4515		0.22 (0.12, 0.32)

MDD – Minimum Dietary Diversity, BCC – Behavior Change Communication, DDS – Dietary Diversity Score, OR – odds ratio, SMD – standardized mean difference, QE – quasi-experimental, cRCT – cluster randomized controlled trial

First, we show summaries by outcome of interest: MDD and DDS. We look at comparisons both between and within studies (‘within studies’ in the sense that studies with separate treatment arms were included). Overall, both across and within studies, there were more reported MDD outcomes than DDS outcomes (6-23.9 months, MDD: n = 9 studies and DDS: n = 6 studies; 6-60 months, MDD: n = 17 studies and DDS: n = 10 studies).

Overall, we find 4 studies of 9 comparisons suggest a benefit in OR for MDD from a sample of n = 5292 and 3 studies of 6 comparisons suggest a benefit in DDS from a sample of n = 4921 from our pooled analysis of nutrition-sensitive agriculture interventions on child diet diversity (6-23.9 months) (**Table 2**). We find 10 of 17 comparisons suggest a benefit in OR for MDD from a sample of n = 8761 and 7 studies of 10 comparisons suggest a benefit in SMD for DDS from a sample of n = 9187 from the pooled analysis on child diet diversity (6-60 months) (**Table 3**).

**Figure 2:**
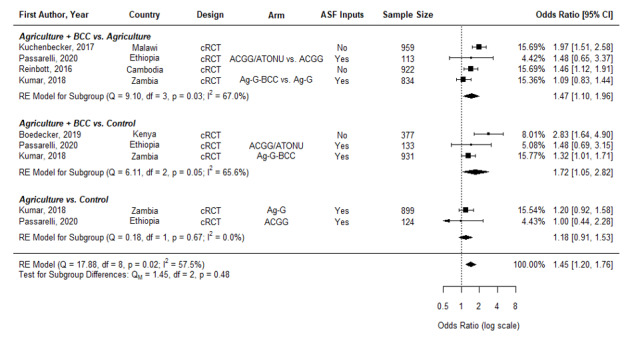
Effects of nutrition-sensitive agricultural interventions on minimum dietary diversity in children aged 6-23.9 months. cRCT – cluster randomized controlled trial, QE – quasi-experimental study, BCC – behavior change communication, OWL – older woman leader, HC – health committee, ACGG – African Chicken Genetic Gains, ATONU – agriculture to nutrition, AG-G – agriculture, gender equity and women’s empowerment, ASF – animal-sourced foods.

**Figure 3:**
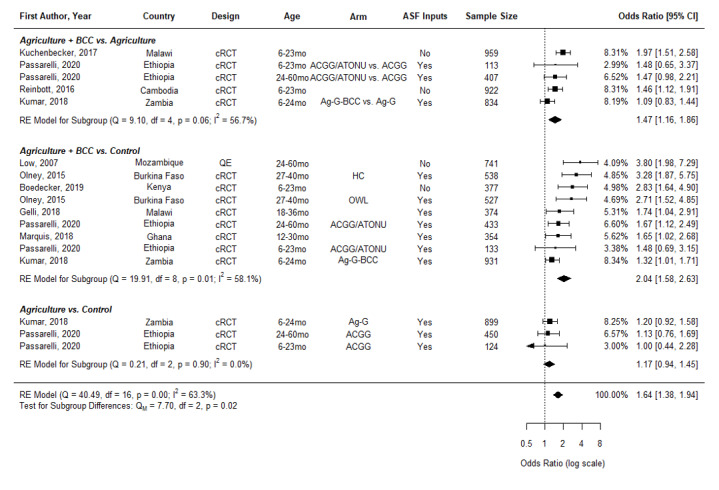
Effects of Nutrition-sensitive Agricultural Interventions on Minimum Diet Diversity in Children Aged 6-60 months. cRCT – cluster randomized controlled trial, QE – quasi-experimental study, BCC – behavior change communication, OWL – older woman leader, HC – health committee, ACGG – African Chicken Genetic Gains, ATONU – agriculture to nutrition, AG-G – agriculture, gender equity and women’s empowerment, ASF – animal-sourced foods

**Figure 2** and **Figure 3** below show the effects of these interventions on MDD by each age grouping.

Nutrition-sensitive agriculture interventions significantly increased the odds of children aged 6-23.9 months reaching minimum diet diversity (OR = 1.45, 95% CI = 1.20-1.76) (**Figure 2**). We then disaggregate into those agriculture interventions including nutrition BCC compared to an agriculture control (Ag + BCC vs Agriculture) and those compared to a naïve control (Ag + BCC vs control, as well as those agriculture arms without nutrition BCC compared to a naïve control (Ag vs Control). We find the odds of reaching minimum diet diversity are greater in interventions with BCC (Ag + BCC vs Ag OR = 1.47, 95% CI = 1.10-1.96; Ag + BCC vs Control, OR = 1.72, 95% CI = 1.05-2.82) than without BCC, which do not have a significant effect (Ag vs Control, OR = 1.18, 95% CI = 0.91-1.53). However, we cannot reject the null that these sub-groups are equal (*P* = 0.48).

We found similar results on MDD when the analyses were expanded to studies that included children in the 6-60-month age range (OR = 1.64, 95% CI = 1.38-1.94) (**Figure 3**). Similar to the analysis for the age group of 6-23.9 months, the odds of reaching minimum diet diversity are higher in those interventions with BCC (Ag + BCC vs Ag, OR = 1.47, 95%CI = 1.16-1.86; Ag + BCC vs Control, OR = 2.04, 95% CI = 1.58-2.63) than those without (Ag vs Control, OR = 1.17, 95% CI = 0.94-1.45). Agriculture intervention arms only as compared to a naïve control did not have a significant effect on MDD.

**Figure 4:**
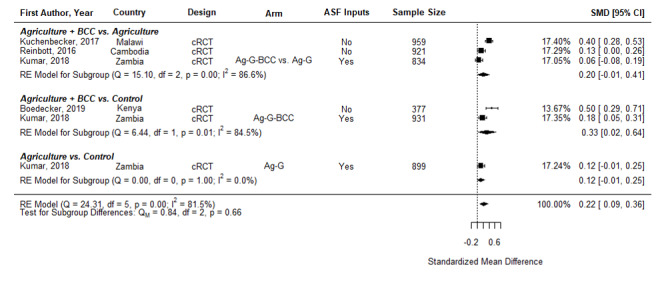
Effects of nutrition-sensitive agriculture interventions on Diet Diversity Score in Children 6-23.9 months. cRCT – cluster randomized controlled trial, QE – quasi-experimental study, BCC – behavior change communication, OWL – older woman leader, HC – health committee, ACGG – African Chicken Genetic Gains, ATONU – agriculture to nutrition, AG-G – agriculture, gender equity and women’s empowerment, ASF – animal-sourced foods.

**Figure 4** presents pooled estimates showing nutrition-sensitive agriculture interventions also significantly increased the standardized mean difference in diet diversity score (DDS) of children aged 6-23.9 months (SMD = 0.22, 95% CI = 0.09-0.36). All point estimates across the subgroups are positive, with Ag + BCC vs a naïve control showing a larger effect (SMD = 0.33, 95% CI = 0.02-0.64) than Ag +BCC vs Ag (SMD = 0.20, 95% CI = -0.01-0.41) or Ag vs Control (SMD = 0.12, 95% CI = -0.01-0.25). The latter two have CIs that narrowly cross zero and are driven by one study [27].

**Figure 5:**
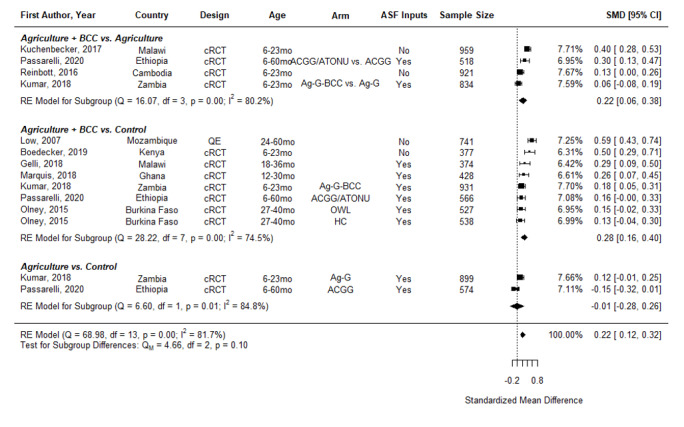
Effects of nutrition-sensitive agriculture interventions on Diet Diversity Score in Children 6-60 months. cRCT – cluster randomized controlled trial, QE – quasi-experimental study, BCC – behavior change communication, OWL – older woman leader, HC – health committee, ACGG – African Chicken Genetic Gains, ATONU – agriculture to nutrition, AG-G – agriculture, gender equity and women’s empowerment, ASF – animal-sourced foods

Further, pooled results show nutrition-sensitive agriculture interventions also significantly increased the standardized mean difference in DDS in children aged 6-60 months (SMD = 0.22, 95% CI = 0.12-0.32) (**Figure 5**). Similarly, Ag + BCC vs a naïve control has a slightly larger effect (SMD = 0.28, 95% CI = 0.16-0.40) than Ag +BCC vs Ag (SMD = 0.22, 95% CI = 0.05-0.38), as might be expected when the control receives some program support. Agriculture interventions without BCC appear to have no positive effect on diet diversity.

### Assessment of study heterogeneity and publication bias

Among the 17 comparisons within 9 studies included in the MDD meta-analysis, the Cochrane Q test was 40.49 (*P* < 0.001) and the *I^2^* proportion was 63.3%. Among the 14 comparisons within 9 studies included in the DDS meta-analysis, the Cochrane Q test was 68.98 (*P* < 0.001) and the *I^2^* proportion was 81.7%. Both suggest substantial heterogeneity between studies and support the use of the random effects model. The trim-and-fill method detected no evidence of publication bias, adding no hypothetical studies to the data set (see Figure S1 in the **Online Supplementary Document** for a funnel plot assessing symmetry of treatment effect estimates).

## DISCUSSION

This study synthesized data on the impacts of nutrition-sensitive agriculture programs on children’s diets. Our results show that nutrition-sensitive agriculture interventions have consistent, positive impacts on dietary diversity of children under 5 years of age in low- and middle-income countries, with no substantive variations in effect sizes observed across the age groups of 6-23.9 and 6-60 months. We primarily focus on the 6-23.9 months age group as the first 1000 days of a child’s life are the critical window for nutrition interventions [19]. We analyzed the two age groups separately because there is not complete overlap in the studies included as some interventions target larger age ranges and because it is useful for modeling. The two age groups showed similar results, although the analysis of the larger age group (6-60 months) included more studies. No studies suggested a benefit in diet diversity from agriculture-only interventions (without a nutrition BCC component). Moreover, the results of the sub-group comparisons are suggestive of the additive benefit of nutrition BCC. As expected, the larger effect sizes are observed between the comparisons with a pure control group. Likewise, decreasing effect sizes are observed in the relative comparisons between agriculture programs with and without BCC.

These results suggest that the BCC components are potentially driving the effect on diets [29]. This is possibly a result of increasing the intent to achieve nutrition-related objectives as well as promoting activities that drive nutrition benefits; two key recommendations from the Lancet review of nutrition-sensitive programs [3]. However, it is hard to pick apart these effects. This is due to the lack of detail on BCC components, lack of standardization of BCC design and insufficient data on implementation fidelity. The range of overall intervention designs and the heterogeneity of BCC component design and implementation make syntheses difficult to interpret. This highlights the need to start from the study design phase to address these issues.

Likewise, heterogeneity is a problem more broadly for the evaluation of multisectoral nutrition-sensitive programs. It is hard to disentangle which components are driving impacts – ie, the effects of individual components vs the effects of the integrated program. The challenge is that nutrition-sensitive programs have different designs, impact pathways, implementation platforms and program activities. In our review, we encountered heterogeneity in evaluation design, duration and enrolment practices – some with repeated cross-sections and others with longitudinal designs. There was also considerable heterogeneity in effect sizes. Similar to meta-analyses conducted for complementary infant and young child feeding practices [30,31], the variability in effect sizes is a result of heterogeneities in program design as well as differences in implementation fidelity and context. Much work is left to be done on intervention design before we can achieve more systematic understanding of the mechanisms driving the effects of nutrition-sensitive agriculture programs.

Future research is needed comparing the interactive effects of these programs – ie, the additive components and synergies between program activities and how they are linked to impacts. Trials of additive programs with multiple treatment arms are valuable as they allow for comparisons within intervention components and would identify any added value of integration. Cross-country studies using these study designs and specifically testing the additive benefits of BCC would be very informative. Although nutrition BCC is widely considered a key component of nutrition-sensitive agricultural interventions [10], without thorough and systematic analysis of BCC approaches, it is difficult to understand the contributions of the nutrition knowledge pathway to these agricultural interventions. Unfortunately, we do not have enough evidence from this analysis to suggest impacts are additive; we find consistent effects on diets but much heterogeneity. However, this review helps refine future questions on whether these interventions have significant additive effects.

This analysis makes an important contribution by thoroughly reviewing the updated state of the evidence on nutrition-sensitive agriculture interventions and producing effect size estimates on the impact of these programs on children’s dietary diversity. How these effects compare to other nutrition-sensitive programs that aim to improve diet diversity - such as cash transfers - remains an open question. A recent review of cash transfers assessed impacts on diet diversity, however, the diet impacts were measured at household level; measuring household food access rather than nutrient adequacy [32]. This is a lacuna in the literature and highlights a need for comprehensive synthesis of nutrition-sensitive programs in general. We did find individual studies of nutrition-sensitive programs and found a few comparable parameters: in Yemen, a study of food and cash transfers found that children in the cash treatment group were 10 percentage points more likely to have a minimally diverse diet (MDD) than who received food [33]. Another study, an RCT in Bangladesh, had five treatment arms: a monthly cash transfer equivalent to 25% of household income; a food basket of equivalent value; a ½ food, ½ cash payment; and cash or food plus nutrition BCC. The cash + BCC arm increased the likelihood that children consumed four or more different food groups by 44 percentage points and the food + BCC arm increased the likelihood by 35 percentage points [34].

Our findings highlight other important areas for future work and provide estimates that could be used in modeling. Currently, most modeling tools are limited in that they do not include multisectoral nutrition-sensitive programs. The current set of nutrition investments included in current models are insufficient to meet the sustainable development goals (ie, a 40% reduction in stunting, 50% reduction in anemia for women) as shown in the 2013 Lancet series [19]. There is a critical need to include a broader set of interventions – such as those that address the underlying determinants of malnutrition - to improve nutrition outcomes into current impact and optimization modeling efforts to inform priority-setting for nutrition investments [35].

The results of this study can be used to provide parameters for modeling tools that estimate the impact of scaling-up nutrition interventions. This process would be a first step to consider these types of interventions for policy decisions, which currently do not allow policymakers to factor in nutrition-sensitive agriculture programs. These parameters could be updated as the evidence on these interventions grows. One example for potential use of these estimates is in the mathematical modeling of intervention coverage, such as with the Lives Saved Tool (LiST). LiST was developed to generate estimates of the impact of changes in coverage for maternal, newborn, child health and nutrition (MNCHN) interventions on cause-specific mortality in low- and middle-income countries (LMICs). LiST calculates changes in mortality due to intervention coverage change, effectiveness and the percentage of cause-specific mortality that the intervention could affect [36].

One approach would be to model improvements in diet diversity with MDD as a diet quality outcome in LiST, doing so through changes in coverage or through direct entry of changes in MDD. This would allow for policy-relevant comparisons of how nutrition-sensitive agriculture affects a given impact pathway relative to other interventions. This would be a step forward despite the limitations which generally apply to LiST and other modeling software. This approach could also theoretically be used to incorporate other outcome measures from nutrition-sensitive interventions. Diet diversity was the easiest outcome to synthesize as most studies measured outcomes consistently and there is a relatively good consensus on the evidence from the impact pathways. However, similar challenges remain in incorporating other outcomes of multisectoral nutrition programs into LiST due to the lack of coherence in design and evaluation and the existence of multiple outcomes or benefits. In particular, the constraints in LiST also entail careful consideration of the overlap of program impact pathways and the risk of double-counting potential effects. For example, an intervention promoting both food supply and demand-side components, such as agricultural and marketing programs with nutrition BCC, would require separation of the impacts of the supply- and demand-side pathways on stunting and/or mortality to avoid double-counting. This accounting of the intermediate effects along the demand and supply pathways is not straightforward.

Careful incorporation of the effects of nutrition-sensitive interventions on the diet diversity of young children in this widely-used software could aid policymakers, implementers and donors in understanding the benefits of these interventions. While nutrition-sensitive agriculture programs do not necessarily have a direct effect on nutrition outcomes, this study shows that dietary improvements are very plausible intermediate results. Program designers and evaluators can be more explicit about this conceptual link. Nutrition-related interventions often measure stunting as the key indicator of success. With the ongoing nutrition transition, the focus has also shifted towards diet quality. This entails also viewing a quality diet as an endpoint. Diet diversity is a proxy of adequacy of nutrient intake, and as such, it is an indicator of diet quality with intrinsic value and benefit.

### Limitations

Evaluation designs of nutrition-sensitive agriculture interventions are heterogenous, which complicates synthesis. Many of the studies included in the meta-analysis were unadjusted comparisons and several only presented endline measures of diet diversity. Also, there was consistent measurement and homogeneity in the definition of food groups within indicators but the recall periods varied (ie, 7-day vs 24-hour recall). Nutrition-sensitive interventions by design aim to improve multiple health and nutrition outcomes. Many outcomes affected by nutrition-sensitive interventions were not analyzed in our approach. This raises the question of how to incorporate benefit streams from nutrition-sensitive programs not captured by this methodology. Further impact pathways could be assessed as the evidence base grows and the effect sizes of other outcomes become available. We also did not examine interventions that were nutrition BCC or education only. This is because our focus was specifically on nutrition-sensitive agriculture programs and prior analyses have covered this area with the intention of incorporation into LiST [30].

## CONCLUSION

Nutrition-sensitive agricultural interventions that include BCC have a significant positive effect on the odds of achieving minimum diet diversity and on improving diet diversity scores for children aged 6-23.9 months and 6-60 months. These estimates demonstrate the contributions of nutrition-sensitive agriculture interventions to diets, as well as show the potential of linking to distal outcomes like stunting. Synthesizing evidence on the impacts of nutrition-sensitive agriculture interventions on child dietary diversity can contribute to decision-making on the relative benefits of nutrition-sensitive interventions with other MNCHN interventions including nutrition-specific programs.

## Additional material


Online Supplementary Document
