# Supplementary material for: Nutrition-sensitive agriculture programs increase dietary diversity in children under 5 years: A review and meta-analysis
Source: J Glob Health. 2022 Feb 19;12:08001. doi: 10.7189/jogh.12.08001 (PMC8849260; doi:10.7189/jogh.12.08001)

Supplementary Materials

Supplemental Figure 1. Funnel plot assessing symmetry of treatment effect estimates

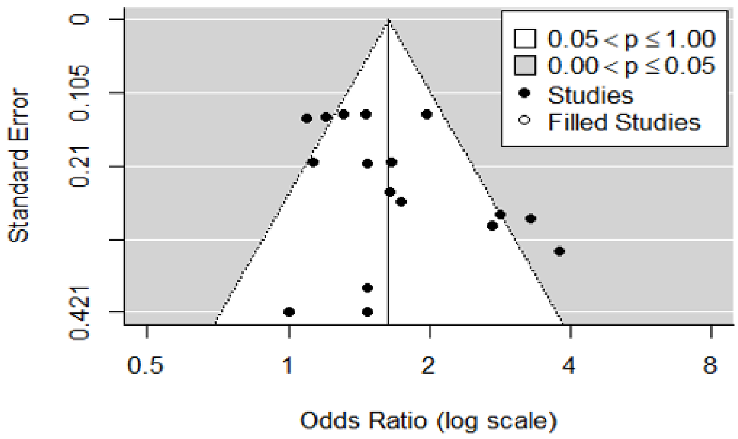

Supplement: Online Supplementary Document [file jogh-12-08001-s001.pdf]
